# Age at surgery and recurrence of ovarian endometrioma after conservative surgery: a meta-analysis including 3125 patients

**DOI:** 10.1007/s00404-020-05586-3

**Published:** 2020-05-19

**Authors:** Fang Yang, Baoqin Liu, Lin Xu, Hong Liu

**Affiliations:** Department of Gynecology of Traditional Chinese Medicine, China-Japan Friendship Hospital, Ying Hua Yuan East Street, Chao Yang District, Beijing, China

**Keywords:** Age, Endometrioma, Meta-analysis, Recurrence, Surgery

## Abstract

**Purpose:**

To evaluate the association between age at surgery and recurrence rate of endometrioma. Data sources PubMed, Embase, and the Cochrane Library were searched up to October 2019.

**Methods:**

We determined the pooled relative risk (RR) and 95% confidence intervals (CIs) to assess the relationship between age at surgery and the recurrence rate of endometrioma after surgery. Begg’s funnel plot and Egger’s linear regression was used to assess any publication bias.

**Results:**

A total of 3125 patients from 10 studies were finally enrolled in this meta-analysis. The recurrence rate decreased with increasing age (RR = 0.93, 95% CI = 0.91–0.95, *P* = 0.451). Subgroup analysis demonstrated that the pooled RR was 0.926 (95% CI 0.906–0.947, *P* < 0.001) for a cut-off < 35, and 0.886 (95% CI 0.775–1.040, *P* = 0.14) for a cut-off ≥ 35. Begg’s funnel plot and Egger’s linear regression test showed no evidence of publication bias.

**Conclusion:**

This meta-analysis suggested that younger age might be a high-risk factor for the recurrence of ovarian endometrioma after conservative surgery.

## Introduction

Endometriosis, characterized by the presence of endometrial glands and stroma outside of the uterine cavity, is responsible for dysmenorrhea, chronic pelvic pain, and infertility [1]. Endometriosis affects approximately 10% of women within their reproductive years [2], resulting in decreased quality of life for the patients and increased healthcare costs. Endometrioma, one of the most common manifestations of endometriosis, is the presence of an ovarian mass arising from the growth of ectopic endometrial tissue in the ovary. The treatment of choice for endometrioma is usually surgery because of the limited efficacy of medication and the possibility of malignancy [3]. Though endometrioma is a benign disease, it can behave malignantly by penetrating and developing in a manner similar to that of cancer metastasis. A frustrating and befuddling aspect of endometriomas is the disease recurrence after surgery. It is known that 21.5% of women will have a recurrence of endometrioma after 2 years and 40–50% within 5 years of surgery [4]. Additionally, some patients require two or more re-operations [5]. And reducing the recurrence rate after surgery is the most difficult problem for clinical practitioners.

Bozdag [6] suggested two hypotheses that seek to explain the underlying pathophysiology of ovarian endometrioma recurrence: growth from residual lesions, or the development of retrograde menstruation after surgery. To prevent the recurrence of endometrioma, various hormonal therapies have been used that function by down-regulating the estrogen level. It is believed that postoperative medical treatment can eradicate microscopic lesions which have not yet been identified and removed surgically [7]. However, with regard to the effects of postoperative medication, there is no consensus. Some studies report that hormonal medications, such as gonadotrophin releasing hormone agonists (GnRH-a), oral contraceptive pills (OCP), and the levonorgestrel-releasing intrauterine system (LNG-IUS), can decrease the recurrence risk of endometriosis [7–9]. While other reports have suggested that the recurrence rate following surgical intervention remains high, even for those receiving postoperative medical therapy, as the hormonal medication can only delay recurrence and not prevent it [10]. Thus, the duration of the postoperative administration should be sufficiently long. A number of studies have been conducted to explore the duration of the follow-up and postoperative medication. Jong et al. suggested that the risk of endometrioma recurrence decreases with age, and after the age of 40, the recurrence rate does not differ according to the use of postoperative medication [11]. Some studies also suggested that a younger age at surgery may lead to a higher likelihood of recurrence [11, 12], but there is no consensus with other studies [13]. Therefore, to help the clinicians apply individual management to achieve better efficacy, we should pay more attention to the relevant factors with the recurrence of ovarian endometrioma after surgery. Therefore, a comprehensive and systematic is necessary understand the association of age at surgery with the recurrence of endometrioma. The aim of the current meta-analysis was to evaluate the association between age at surgery and the recurrence rate of endometrioma after conservative surgery.

## Materials and methods

### Search strategy

For this meta-analysis, we carefully and systematically searched PubMed, Embase, and the Cochrane Library for relevant studies published online before October 2019. The search terms included: “endometriosis” (e.g., “endometrioses”, “endometriomas”) and “recurrence” (e.g., “relapse”, “recrudescence”). References in the retrieved articles were also manually searched for additional studies.

### Inclusion and exclusion criteria

We included the following studies for this meta-analysis: (1) studies performed on humans; (2) studies patients of ovarian endometrioma confirmed by pathological examination that were treated with robot-assisted surgery or traditional approaches (laparoscopy or/and laparotomy); (3) studies in which ultrasound was conducted to determine the endometrioma recurrence at least 6 months after conservative surgery; and (4) studies that reported a correlation between age at surgery and recurrence of endometrioma. We excluded (1) abstracts, letters, case reports, reviews or nonclinical studies; (2) studies with insufficient data for estimating the HR (hazard ratio), RR (relative risk) and 95% confidence interval (CI); (3) studies that were not written in English; and (4) studies that had duplicate data or repeat analysis.

### Data extraction and quality assessment

All candidate articles were independently evaluated and extracted by the two authors (Fang Yang and Baoqin Liu). If the articles could not be categorized by the title and abstract alone, then the full-text reviews were retrieved. If disagreement occurred, the two authors discussed and arrived at a consensus with a third author (Hong Liu). The following items were extracted from eligible studies: first author, year of publication, country, number of patients, treatment strategy, follow-up, r-ASRM stage of endometrioma, endometrioma size, and RRs with 95% CIs. The Newcastle–Ottawa Scale (NOS) was used by two independent authors (Fang Yang and Baoqin Liu) to assess the quality of each of the included studies. The NOS consists of three parts: selection (0–4 points), comparability (0–2 points), and outcome assessment (0–3 points). NOS scores of ≥ 6 were considered to be high-quality studies.

### Statistical analysis

This meta-analysis was performed with STATA statistical software (version 12.0; College station, TX). All the results for the binary outcomes that were obtained from the literature are shown as RR/HR and 95% CI (confidence interval). A RR/HR < 1 suggested a preventive value in the recurrence of endometrioma with age at surgery. Cochran’s *Q* test and Higgins, I-squared statistic were undertaken to assess the heterogeneity of the included trials. When *P* > 0.05 OR *I*^2^ ≤ 50%, the fixed effects (Mantel–Haenszel method) model was used, because it indicated acceptable heterogeneity. Otherwise, when *P* < 0.05 or *I*^2^ > 50%, a random effects model was used, which suggested significant heterogeneity in the literature. Subgroup analysis was conducted to explore and explain the diversity (heterogeneity) among the results of different studies. Publication bias was assessed by Begg’s funnel plot and Egger’s linear regression test. All *P* values were two-sided, and a *P* value < 0.05 was considered statistically significant.

## Results

### Literature search

**Fig. 1:**
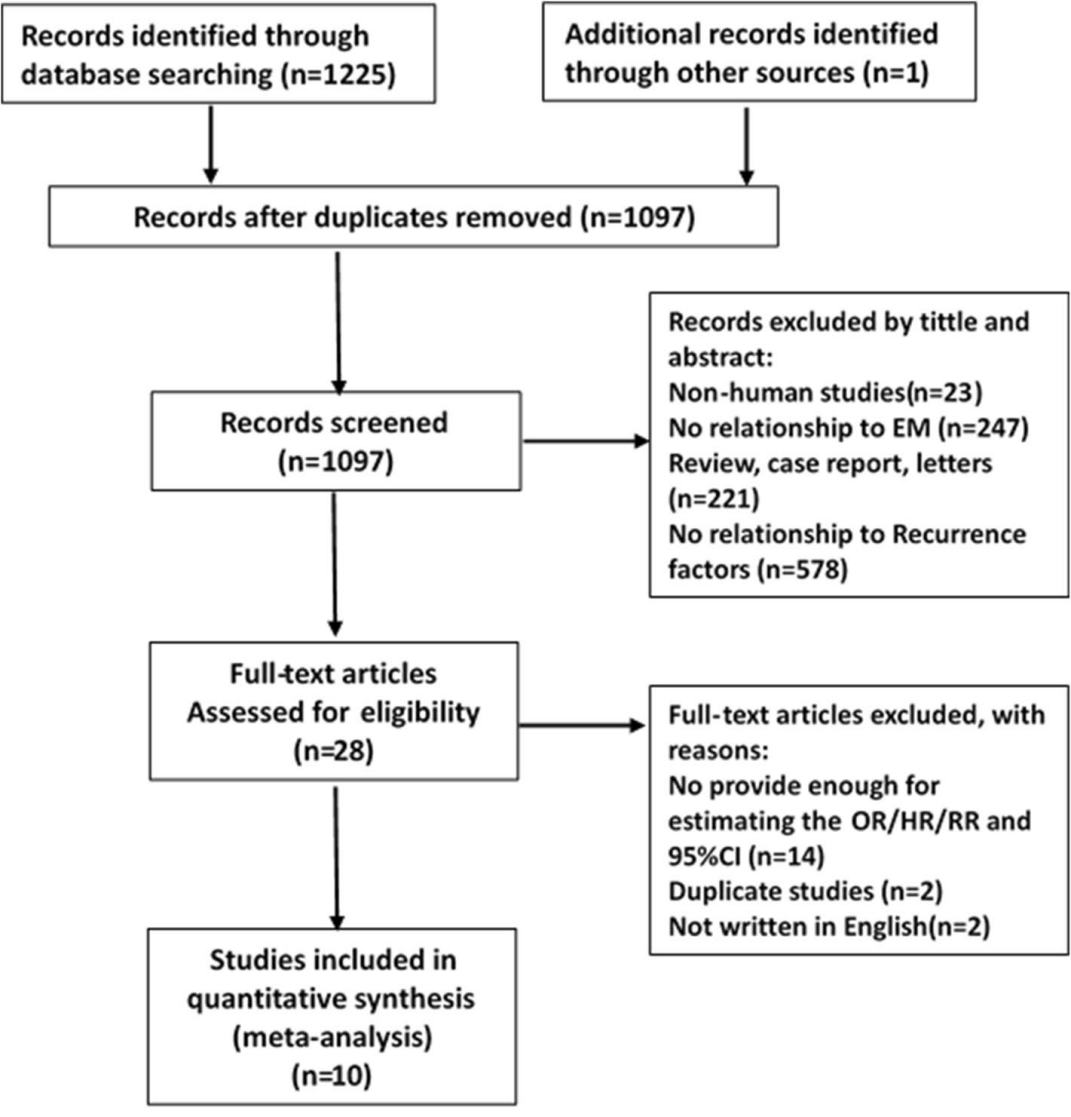
The flowchart showed the selection of studies for meta-analysis

A total of 1226 related studies were initially retrieved. After excluding 129 duplicate studies, 1097 studies were screened by 2 authors. In total, 1069 studies were excluded after title and/or abstract screening for the following reasons: non-human studies (*n* = 23), studies with no relationship to endometriosis (*n* = 247), abstracts, letters, case report, review or nonclinical studies et al. (*n* = 221), and no relationship with recurrence or recurrence factors (*n* = 578). Thereafter, the full text of 28 studies was assessed; 14 studies were excluded because of insufficient data (*n* = 14), and 2 studies were excluded due to duplication and not written in English (*n* = 2). A total of ten studies that met our selection criteria were finally included in this meta-analysis (14–23). The detailed selection process is presented in Fig. 1.

### Study characteristics

After meticulous inspection of the articles, 10 studies with a total of 3125 patients, published between 2007 and 2019 were finally enrolled in this meta-analysis. The characteristics of the included studies are shown in Table1. The RRs and 95% CIs were reported directly in eight studies (14, 16–20, 22, 23), and in the other two studies, the RRs and 95% CIs were obtained by OR transformation (15, 21). Among these, three studies were from China (14, 19, 23), three were from Japan (16, 20, 22), three were from Korea (15, 17, 18), and the last one was performed in Italy (21). In these studies, the surgical procedure was laparoscopy in six cases (14, 17, 18, 20–22), whereas the others were laparoscopy and/or laparotomy (15, 16, 19, 23). In total, five studies involved all stages of r-ASRM (I–IV) (14, 15, 17, 21, 23), three studies included only late stage (III–IV) (18–20), and two studies did not provide any information on the r-ASRM stage (16, 22). There were two prospective studies (19, 22) and the remaining eight studies were retrospective (14–18, 20, 21, 23). The NOS scores ranged from 6 to 8 and the mean was 7.10; this indicated that all included studies were of high quality.

**Table 1:** Main characteristics of all the studies included in the meta-analysis

Study cohort	Year	Study region	ethnicity	Follow-up (months)	Sample size	Age	r-ASRM stage		Endometrioma size (cm)		RR	Surgery type	NOS
							I–II (*n*)	III–IV (*n*)	Non-recurrence	recurrence			
Xiao-Yan [14]	2019	China	Asian	82	358	33	11	347	5.4 ± 2.2	5.6 ± 1.9	R (M)	Laparoscopy	7
Sieun [15]	2018	Korea	Asian	57	134	34	11	123	NR	NR	R (U)	Combine	6
Tobiume [16]	2016	Japan	Asian	29	352	NR	NR	NR	6.2 ± 2.7	7.2 ± 3.2	R (M)	Combine	7
BoHyon Yun [17]	2015	Korean	Asian	27	379	29	8	371	4.9 ± 2.4	5.5 ± 2.6	R (M)	Laparoscopy	7
Sihyun [18]	2014	Korean	Asian	17	99	38	0	99	4.2 ± 2.1	4.5 ± 2.2	R (U)	Laparoscopy	7
Ming [19]	2014	China	Asian	29	307	32	0	307	5.6 ± 2.8	6.7 ± 2.5	P (M)	Combine	7
Kazuo [20]	2013	Japan	Asian	60	248	31	0	248	5.7 ± 1.9	5.4 ± 1.7	R (M)	Laparoscopy	8
Maria [21]	2011	Italy	Caucasian	72	401	33	114	287	NR	NR	R (M)	Laparoscopy	8
Takamura [22]	2009	Japan	Asian	24	137	33	NR	NR	NR	NR	P (U)	Laparoscopy	7
XiShi Liu [23]	2007	China	Asian	22	710	36	117	593	NR	NR	R (M)	Combine	7

*RR* relative risk, “*M*” means the RR come from multivariate analysis, “*U*” means the RR from univariate analysis, *Combine* combine with the laparoscopy and laparotomy, *NR* not report, *NOS* Newcastle–Ottawa Quality Assessment Scale

### Relationship between age at surgery and recurrence of ovarian endometrioma

**Fig. 2:**
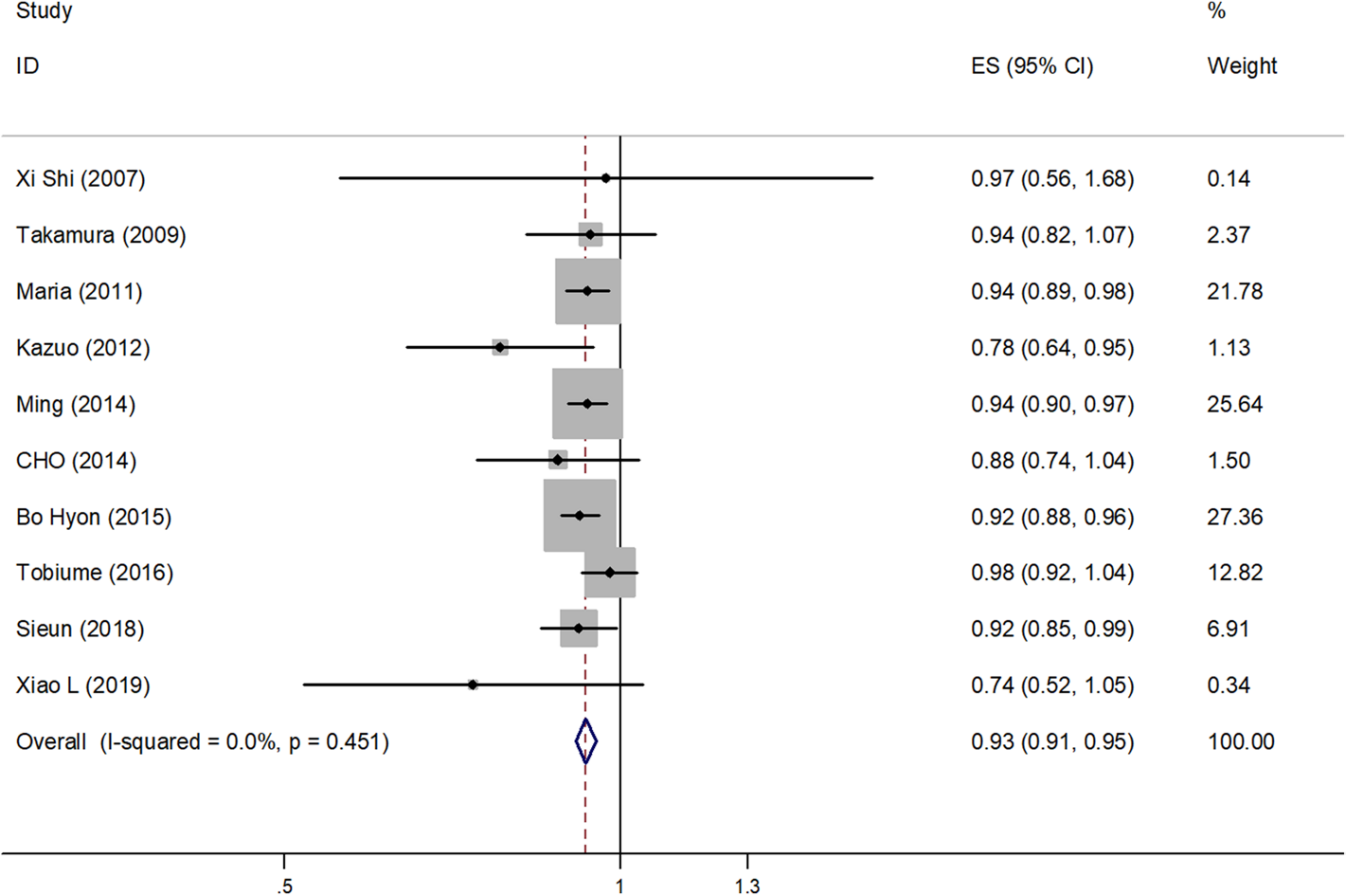
Forest plot diagrams of relative risk for correlations between age at surgery and recurrence rate

In total, 10 studies (14–23) with 3125 patients reported the relationship between age at surgery and the recurrence rate of ovarian endometrioma after conservative surgery; the pooled RR and 95% CI were determined to explore the correlation. Since the heterogeneity was not significant (*I*^2^ = 0.0%, *P* = 0.451), a fixed-effects model was applied. Our results revealed that the recurrence rate of endometrioma decreased with increasing age (RR = 0.93, 95% CI = 0.91–0.95, *P* < 0.001) (Fig. 2), demonstrating a clear time trend of age-related recurrence.

**Fig. 3:**
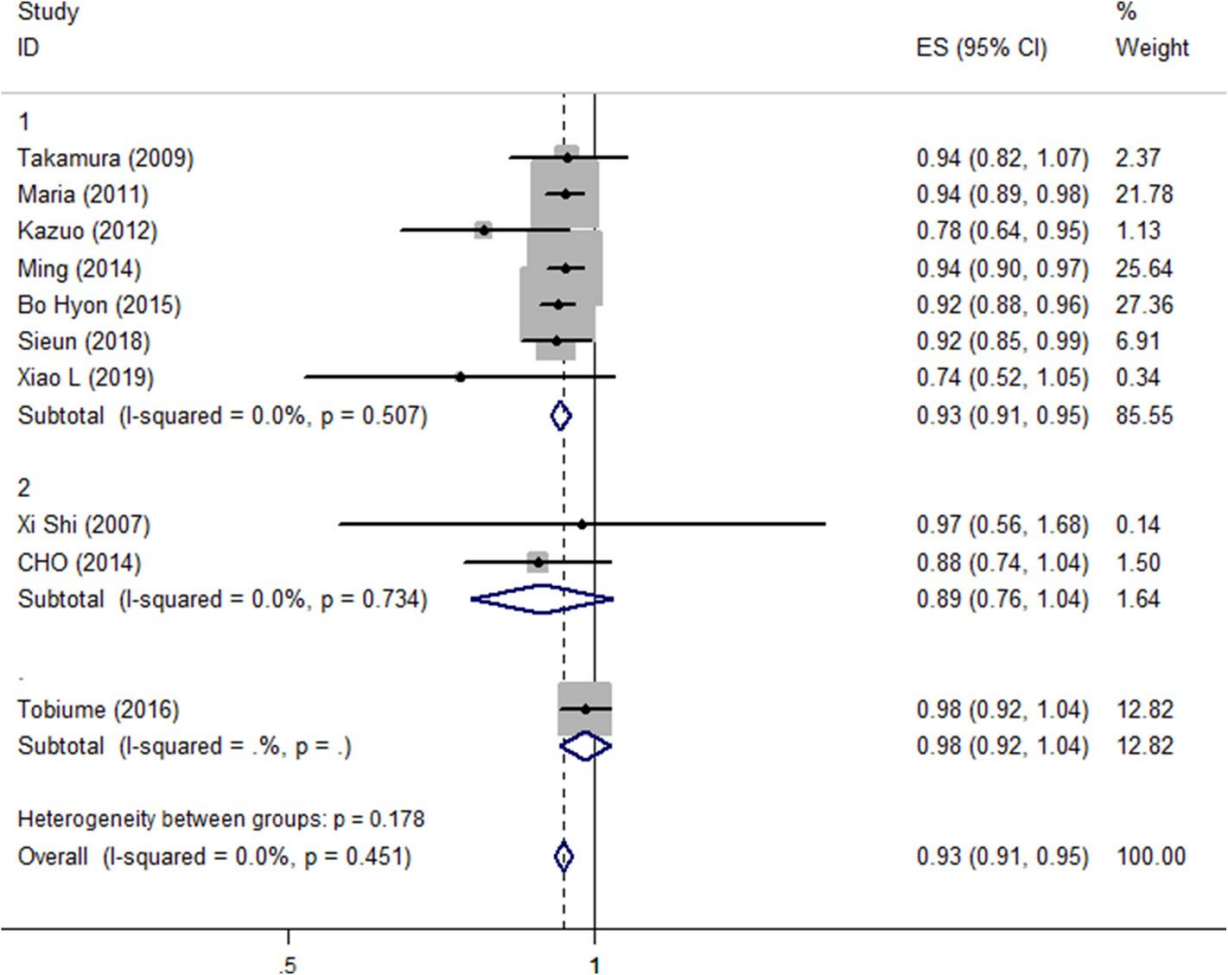
Forest plot diagrams of relative risk for correlations between age at surgery and recurrence rate (subgroup: age ≥ 35 years; age < 35 years)

Subgroup analysis was performed because several of the baseline characteristics varied among the included studies. We analyzed age at surgery < 35 years, and age ≥ 35 years, and the data demonstrated that the pooled RR was 0.926 (95% CI 0.906–0.947, *P* < 0.001) for age < 35 years, and 0.886 (95% CI 0.775–1.040, *P* = 0.14) for age ≥ 35 years (Fig. 3). In addition, subgroup analysis was performed by the r-ASRM stage, surgery type (laparoscopy and laparoscopy/laparotomy) and univariate analysis and multivariate analysis (Table 2).

**Table 2:** Summary of the meta-analysis results

Analysis	*N*	Random-effects model			Fixed-effects model		Heterogeneity	
		References	RR/HR (95% CI)	*P*	RR/HR (95%)	*P*	*I*^2^	Ph
Recurrence	10	[14–23]	0.932 (0.913–0.951)	0	0.932 (0.913–0.951)	0	0%	0.451
Age (years)								
≥ 35	2	[18, 23]	0.886 (0.775, 1.040)	0.14	0.886 (0.775, 1.040)	0.14	0%	0.734
< 35	7	[14–17, 19–21]	0.926 (0.906, 0.947)	0	0.926 (0.906, 0.947)	0	0%	0.507
r-ASRM stage								
I–IV	5	[14, 15, 17, 21, 23]	0.934 (0.911, 0.957)	0.021	0.934 (0.911, 0.957)	0	0.20%	0.414
III–IV	3	[18–20]	0.892 (0.870, 0.983)	0	0.925 (0.890, 0.961)	0	45.20%	0.161
Surgery type								
Laparoscopy	6	[14, 17, 18, 20–22]	0.921 (0.864, 0.982)	0.012	0.940 (0.909, 0.972)	0	49.80%	0.093
Laparoscopy/laparotomy	4	[15, 16, 19, 23]	0.927 (0.903, 0.951)	0	0.927 (0.903, 0.951)	0	0%	0.978
Analysis								
Univariate analysis	3	[15, 18, 22]	0.903 (0.851, 0.951)	0.001	0.903 (0.851, 0.951)	0.001	0%	0.412
Multivariate analysis	7	[14, 16, 17, 19–21, 23]	0.936 (0.915, 0.956)	0	0.936 (0.915, 0.956)	0	0%	0.441

### Publication bias

**Fig. 4:**
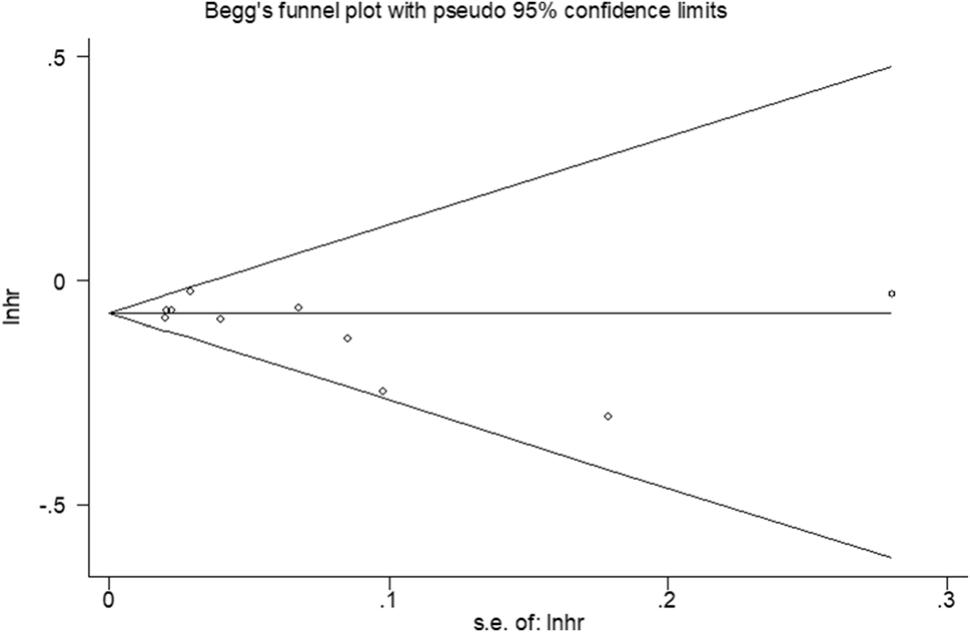
Funnel plots for publication bias

Begg’s funnel plot and the Egger’s linear regression test were performed to evaluate publication bias. Begg’s tests showed that there was no publication bias in the included studies, pr > │z│ = 0.180 (Fig. 4). Similarly, Egger’s tests, the publication bias was also not detected, and *P* > │t│ = 0.171.

## Discussion

Although a large number of studies have investigated the association of age with the recurrence of ovarian endometrioma after conservative surgery, the results have been inconsistent and inconclusive. Therefore, we reviewed the published studies and undertook a meta-analysis to derive a more precise estimation of the relationship between age at surgery and recurrence. Our meta-analysis combined the outcomes of 3125 endometrioma patients after surgery from ten individual studies, and indicated that younger age significantly predicted high recurrence rate (RR = 0.932, 95% CI 0.913–0.951, *P* < 0.001). Subgroup analysis revealed that the risk of endometrioma recurrence after surgery decreased with increasing age, in ages < 35 years old (RR = 0.926, 95% CI = 0.906–0.947, *P* < 0.001). In contrast, in ages ≥ 35 years, there was no association with recurrence (RR = 0.886, 95% CI = 0.775–1.040, *P* = 0.14). Additionally, subgroup analyses showed that younger age was associated with a higher risk of recurrence for several of the surgeries including laparoscopy and laparotomy. And regardless of the r-ASRM stage, the risk of recurrence decreased with age. However, of the ten studies, there was no RR/HR for the early stage (I–II), separately. Therefore, in the subgroup meta analyses (shown in Table 2), the r-ASRM I–IV were represented together versus stage III and IV, rather than the early stages (I–II) versus the advanced stages (III–IV). There is an absence of sufficient evidence to provide the relationship between the r-ASRM stage and the recurrence rate. Taken together, the age at surgery maybe significantly associated with the risk of endometrioma recurrence.

Endometrioma causes impaired quality of life for women of reproductive age, and has malignant clinical manifestation despite being a benign disease. Recurrence is one of the main problems for ovarian endometriomas after conservative surgery. The relationship between the patients’ age at surgery and endometrioma recurrence has been consistently mentioned in previous studies. Some previous studies reported that younger age represents a determinant for recurrence [11, 20, 24]. In these studies, it was hypothesized that the estrogen production and the circulating estrogen levels decrease, as women age; therefore, younger age is likely to be characterized by higher circulating levels of estrogen, which may go some way to explain the high rate of recurrence. In addition, younger age at surgery would also signify a younger age at onset of ovarian endometrioma, and younger age at onset may represent a disease form that may be different to that observed in women with later onset; it is conceivable that this different form may be more aggressive and prone to recurrence [23, 24]. Seo et al. [11] reported that, the cumulative endometrioma recurrence rates were 43.3% for patients aged 20–29 years, 22.5% for patients aged 30–39 years, and 10.2% for patients aged 40–45 years. Xiao-Yan et al. [14], also showed that the recurrence rate decreased with increasing age. However, Parazzini et al. [25] suggested that the endometrioma recurrence rate tended to increase with age and reported a recurrence rate of 4.6% among women aged 20–30 years, and 13.1% among women aged > 30 years. Our meta-analysis revealed the association between age at surgery and the recurrence rate of ovarian endometrioma after surgery by demonstrating that the younger age at surgery, the higher the risk of the recurrence (RR = 0.932, 95% CI 0.913–0.951, *P* < 0.001). Otherwise, after the age of 35, the recurrence rate did not differ (RR = 0.886, 95% CI = 0.775–1.040, *P* = 0.14).

There are several limitations of the current meta-analysis that should be considered when interpreting the findings. First, because most of the included studies were retrospective in design, the analysis may have a selection bias with regards to patient characteristics. Second, the majority of the eligible studies were conducted in Asia, which may reflect the situation in Asia only; thus, our results may not be generalizable to other patients in other parts of the world. Third, since the analysis was constrained to studies published in English language only, a publication bias cannot be excluded. Another limitation is that the recurrence of endometrioma after surgery was evaluated using ultrasound instead of surgery with histological confirmation.

## Conclusion

In conclusion, we have documented that younger age (especially < 35 years) at surgery is a significant risk for recurrence of ovarian endometrioma after conservative surgery. We believe that our findings are helpful for counseling patients at high risk of recurrence. This information suggests that gynecologists performing conservative surgery should be aware of patients’ age, pay close attention to the follow-up of younger patients and plan the postoperative management strategy appropriately in order to reduce the risk of recurrence. However, the studies (age ≥ 35 years) that we searched were only two, so the sample size of age at surgery (≥ 35) was too small to assess the relationship age ≥ 35 years with the recurrence rate. The meta-analysis should be updated if more large-scale controlled studies are published, especially the recurrence rate of patients aged ≥ 35 years.
